# Pacemaker Pocket Stabilization Utilizing a Novel Envelope and a Three-Point Anchoring Technique

**DOI:** 10.7759/cureus.13108

**Published:** 2021-02-03

**Authors:** Naga Venkata K Pothineni, Ramanan Kumareswaran, Robert D Schaller

**Affiliations:** 1 Cardiac Electrophysiology, Hospital of the University of Pennsylvania, Philadelphia, USA

**Keywords:** pacemaker pocket infection, pacemaker complication, twiddler's syndrome

## Abstract

A 65-year-old man presented with chronic pain due to frequent movement of a dual chamber pacemaker (PPM) within the device pocket despite being secured to the underlying muscle. Due to chronic pain and possible indolent infection, the PPM was removed and a new device was implanted on the contralateral side via a persistent left superior vena cava. To prevent device movement, it was placed within a CanGaroo® envelope (Aziyo Biologics Inc., Silver Spring, MD, USA), which was secured to the underlying muscle with a silk suture along three of its corners. The envelope, which becomes incorporated into the surrounding tissue forming a vascularized tissue pocket, should further reinforce device stability over time. The patient’s left-sided symptoms abated immediately and he remains free of symptoms on the right side over a six-week follow-up period.

## Introduction

Cardiac implantable electronic devices (CIED) are a well-established treatment modality for pacing and prevention of sudden cardiac death. The last two decades have seen an exponential increase in CIED implantation rates, with a concomitant rise in device-associated complications [1]. While systemic bacteremia and endocarditis represent extreme forms of device-related infection, subacute presentations of indolent CIED pocket infection present a clinical challenge. Chronic discomfort over the CIED generator site is not uncommon and can be due to frequent movement or rotation of the device around multiple axes [2]. In this report, we present a case of chronic CIED device site discomfort and propose a novel solution.

## Case presentation

A 65-year-old male was referred for evaluation of chronic pain over his pacemaker (PPM) site. He had a history of chronic obstructive lung disease, diabetes mellitus, and aortic stenosis previously treated with a bioprosthetic aortic valve two years prior. His post-operative course was complicated by complete heart block requiring a dual-chamber PPM implanted on the right due to a persistent left superior vena cava (SVC) draining into the coronary sinus. Upon follow-up, he reported persistent discomfort at the PPM site exacerbated by positional changes and movement of the right shoulder. He also reported device migration and rotation within the pocket, with an ability to flip the device around the X and Z axes [2]. On examination, the device site had no signs of active infection. The generator was palpable and easily rotated within the CIED pocket around the single suture, with reproducible pain during generator movement. Given the significant impact of symptoms on his quality of life and possibility of an indolent infection, the patient presented to the electrophysiology laboratory for extraction of the existing PPM system and re-implantation on the contralateral side.

**Figure 1:**
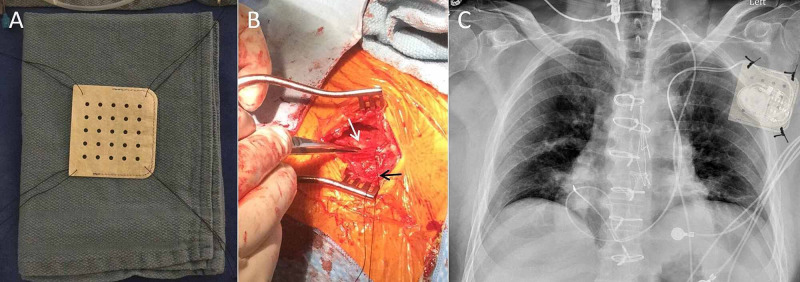
CanGaroo® envelope pocket securement technique. (A) Silk sutures have been placed in all four corners of the envelope to demonstrate securement options. (B) The PPM has been placed in the CanGaroo® envelope (white arrow) and a silk suture (black arrow) has been placed in one corner. (C) Anterior-posterior chest X-ray of a dual-chamber PPM via a left persistent SVC with an image overlay of the CanGaroo® envelope and anchoring sutures representative of a three-point securement technique. PPM, pacemaker; SVC, superior vena cava

Intra-operatively, the device was found to be anchored to the pectoral muscle with no clear evidence of pocket infection. The generator was mobile and easily movable around both the X and Z axes within the pocket. The leads and generator were freed up and extracted from the vasculature with simple traction. After the patient was re-prepped and the operators re-scrubbed, a new dual-chamber PPM system was implanted on the left side via the left axillary vein and persistent left SVC (Figure 1C). Prior to insertion in the pocket, the generator was placed in a CanGaroo® envelope (Aziyo Biologics Inc., Silver Spring, MD, USA), which had first been soaked in an antibiotic solution containing neomycin and polymyxin B. The device and envelope were then anchored to the pectoral muscle by a three-point fixation to prevent all movement within the pocket (Figure 1A, 1B). Post-operatively, the patient’s right-sided discomfort immediately abated, and there has been no device movement or significant symptoms related to the left-sided PPM system over a six-week follow-up period.

## Discussion

Chronic pain at a CIED site is an infrequent, yet challenging complication encountered in clinical practice with multiple possible etiologies. While chronic indolent infection and regional pain syndromes are often considered and treated, generator movement within the pocket is often overlooked. Prior studies have shown that almost a quarter of patients undergoing CIED implantation report pain or discomfort with movement of the generator in the pocket [3]. Shoulder pain and limitation of movement ipsilateral to the CIED site are also common [4]. Management of these conditions presents a clinical dilemma with attempts to balance patient comfort and increased risk of infection with subsequent pocket revisions.

Subclinical infection and improper surgical technique with poorly formed pockets have been found to be the major etiologies for chronic pain [5], consistent with previous reports [6]. A recent multi-center study evaluating outcomes of lead extraction for chronic pain showed significant improvement in overall symptoms with removal of the device. While percutaneous lead extraction represents a reasonable treatment strategy and carries a class IIa indication for system extraction for management of chronic pain [7], it is associated with increased risk depending on patient-specific features [8]. Due to our patient’s ongoing discomfort, unknown infectious status, and short lead dwell time, we decided to remove his leads and place a new PPM on the contralateral side, despite the challenges of a persistent left SVC.

Attention to optimal CIED pocket management techniques during the initial implant procedure can decrease the risk of both indolent infection as well as device migration, both of which are major causes of chronic pain. In this context, the CanGaroo® envelope, a novel decellularized extracellular matrix derived from porcine small intestine submuscosa, is an appealing option [9]. The affixed envelope becomes incorporated into the surrounding tissue, forming a vascularized tissue pocket that maintains the anchoring sutures in place and reinforces them over time, which is not possible with similar envelopes that are absorbable. This enhanced stability may be important in the context of Twiddler’s syndrome or with devices that tend to flip or rotate spontaneously, occasionally around a single anchoring site, the standard on all commercially available PPMs.

Whether the CanGaroo® envelope is useful in preventing CIED-related pocket infections requires further investigation. Decellularized extracellular matrix supports proliferation functions by attracting stem cells, stimulating angiogenesis, and altering the immune response by eliciting an M2 regenerative response [9]. Additionally, pre-clinical studies have been promising showing prevention of Staphylococcus species growth in vitro and substantially reduced incidence of CIED pocket infections in an in vivo rabbit model due to an early antibiotic bolus release and slow elution lasting up to six days.

## Conclusions

Use of a CanGaroo® envelope with a multi-point anchoring strategy represents an appealing option for CIED pocket stabilization to prevent movement of the device within the pocket. This strategy should also address rotational device movement, an under-recognized phenomenon that can lead to patient discomfort. Future research should concentrate on infection prevention considering the antimicrobial properties intrinsic to the novel envelope in addition to its antibiotic elution qualities.
